# 

**Published:** 1871-06

**Authors:** 


### CONTENTS:

ORIGINAL COMMUNICATIONS:

ARTICLE I. Some Remarks upon Cases of Obstruction of the Bowels in which Surgical Means were used. By A. Reeves Jackson, M.D. 321

ART. II. Excisions of Head of Humerus for Gun Shot Wounds. By Charles M. Clark, M D., late Surgeon 39th Ill. Vol. Infantry, and Chief Operating Surgeon 24th Army Corps ...................... 326

ART. III. Reply to the "Criticism" of Dr. H. W. Boyce in Relation to an Article by the Writer upon "Cases in Practice, with Remarks on the use of Veratrum Viride." By B. O. Reynolds, M.D .......... 331

ART. IV. A Case of Non-Union of Fracture of the Femur, caused by the interposition of Muscle between the fragments. By C. T. Parkes, M.D ...................... 338

ART. V. St. Luke's Hospital. Typhoid Fever, treated with Strychnia. Under care of Jno. E. Owen, M.D ............................... 341

ART. VI. Hydrate of Chloral as a Remedial Agent — Effects of its Continued Use. By * * C..... 344

ART. VII. Case of Malposition of the Bladder. By E. Dooley, M.D. 347

SELECTIONS ............................. 349

Proceedings of the Annual Convention of the Am. Med. Association, held at San Francisco, May 2, 1871 ...... 356

CORRESPONDENCE ........................ 367

EDITORIAL ........................... 370

LOOT ................................. 376

NOTICES .............................. 383

